# Supplementary material for: Iterative improvement in the automatic modular design of robot swarms
Source: PeerJ Comput Sci. 2020 Dec 7;6:e322. doi: 10.7717/peerj-cs.322 (PMC7924708; doi:10.7717/peerj-cs.322)
Supplement: Supplemental Information 3 [file peerj-cs-06-322-s003.zip › argos3/doc/api/standalone/a00460_source.html]

ARGoS: plugins/robots/foot-bot/simulator/footbot\_distance\_scanner\_equipped\_entity.cpp Source File


- Main Page
- Related Pages
- Namespaces
- Classes
- Files

- File List
- File Members

# plugins/robots/foot-bot/simulator/footbot\_distance\_scanner\_equipped\_entity.cpp

Go to the documentation of this file.

```
00001 
00007 #include "footbot_distance_scanner_equipped_entity.h"
00008 #include <argos3/core/simulator/physics_engine/physics_engine.h>
00009 #include <argos3/core/simulator/space/space.h>
00010 
00011 namespace argos {
00012 
00013    /****************************************/
00014    /****************************************/
00015 
00016    CFootBotDistanceScannerEquippedEntity::CFootBotDistanceScannerEquippedEntity(CComposableEntity* pc_parent) :
00017       CEntity(pc_parent),
00018       m_unMode(MODE_OFF),
00019       m_fRotationSpeed(0.0f) {
00020       Disable();
00021    }
00022    
00023    /****************************************/
00024    /****************************************/
00025 
00026    CFootBotDistanceScannerEquippedEntity::CFootBotDistanceScannerEquippedEntity(CComposableEntity* pc_parent,
00027                                                                   const std::string& str_id) :
00028       CEntity(pc_parent, str_id),
00029       m_unMode(MODE_OFF),
00030       m_fRotationSpeed(0.0f) {
00031       Disable();
00032    }
00033    
00034    /****************************************/
00035    /****************************************/
00036 
00037    void CFootBotDistanceScannerEquippedEntity::Reset() {
00038       m_unMode = MODE_OFF;
00039       m_cRotation = CRadians::ZERO;
00040       m_fRotationSpeed = 0.0f;
00041    }
00042 
00043    /****************************************/
00044    /****************************************/
00045 
00046    void CFootBotDistanceScannerEquippedEntity::Update() {
00047       if(m_unMode == MODE_SPEED_CONTROL &&
00048          m_fRotationSpeed != 0.0f) {
00049          m_cRotation += CRadians(m_fRotationSpeed *
00050                                  CPhysicsEngine::GetSimulationClockTick());
00051          m_cRotation.UnsignedNormalize();
00052       }
00053    }
00054 
00055    /****************************************/
00056    /****************************************/
00057 
00058    void CFootBotDistanceScannerEquippedEntity::SetRotation(const CRadians& c_rotation) {
00059       m_cRotation = c_rotation;
00060       m_cRotation.UnsignedNormalize();
00061    }
00062 
00063    /****************************************/
00064    /****************************************/
00065 
00066    REGISTER_STANDARD_SPACE_OPERATIONS_ON_ENTITY(CFootBotDistanceScannerEquippedEntity);
00067 
00068    /****************************************/
00069    /****************************************/
00070 
00071 }
```

---

Generated on 10 Jul 2018 for ARGoS by 
 1.6.1
